# The Pupillary Response to the Unknown: Novelty Versus Familiarity

**DOI:** 10.1177/2041669519874817

**Published:** 2019-09-07

**Authors:** Steve Beukema, Ben J. Jennings, Jay A. Olson, Frederick A. A. Kingdom

**Affiliations:** McGill Vision Research, Department of Ophthalmology, Montreal General Hospital, Montreal, Quebec, Canada; Centre for Cognitive Neuroscience, Department of Life Sciences, College of Health and Life Sciences, Brunel University London, UK; Department of Psychiatry, McGill University, Montreal, Quebec, Canada; McGill Vision Research, Department of Ophthalmology, Montreal General Hospital, Montreal, Quebec, Canada

**Keywords:** object, form, recognition, pupillometry, pupil, dilation, constriction, novelty, familiarity

## Abstract

Object recognition is a type of perception that enables observers to recognize familiar shapes and categorize them into real-world identities. In this preregistered study, we aimed to determine whether pupil size changes occur during the perception and recognition of identifiable objects. We compared pupil size changes for familiar objects, nonobjects, and random noise. Nonobjects and noise produced greater pupil dilation than familiar objects. Contrary to previous evidence showing greater pupil dilation to stimuli with more perceptual and affective content, these results indicate a greater pupil dilation to stimuli that are unidentifiable. This is consistent with the relative salience of novelty compared to familiarity at the physiological level driving the pupil response.

## Introduction

Pupil size changes are reflexive; the pupil not only grows and shrinks unconsciously in response to varying levels of light (Ellis, 1981), but it also changes in size depending on the content of the stimulus. This cognitive influence on the pupillary response has been long established (Beatty & Lucero-Wagoner, 2000; Loewenfeld, 1958), but its precise nature is less clear (Mathôt, 2018). In this article, we focus on the pupillary responses to object recognition, in order to deepen our understanding of the cognitive influences on the pupillary response as well as on object recognition itself.

Object recognition is an important part of human perception, necessary not only for determining the content of the visual world but also to establish links between familiar identities in our past, present, and future. One issue that engages researchers using pupillometry to study object recognition concerns the process of distinguishing familiar from nonfamiliar objects (DiCarlo, Yoccolan, & Rust, 2012; Grill-Spector, 2003; Kafkas & Montaldi, 2015). For example, Kafkas and Montaldi (2015) showed subjects a range of familiar objects during a training phase and then measured the pupil responses to a set of test objects, some of which had been presented in the training phase. They found that the test objects that had been seen during the training phase produced a greater pupil dilation than the ones not previously seen. In a similar study by Naber, Frassle, Rutishauser, and Einhauser (2013), subjects viewed images of everyday scenes and were asked to memorize half of them. After a distraction phase, the images were presented again but intermixed with novel images, and the subjects indicated whether the images were familiar or novel and how confident they were in their judgement. Consistent with the results of Kafkas and Montaldi (2015), a greater pupil dilation was observed for the memorized scenes, while the novel scenes produced a pupil constriction that became larger as a function of confidence.

Unlike Kafkas and Montaldi (2015), however, Naber et al. (2013) tested the pupil response during both retrieval *and* encoding. Interestingly, a greater constriction response was observed during the encoding phase for scenes that were later remembered compared to the scenes that were later forgotten. Then, during the retrieval phase, the novel (i.e., unfamiliar) scenes produced a greater constriction compared to the familiar (i.e., remembered) scenes. The authors thus showed that under the task of retrieving a memory, stimulus novelty could produce a pupil *constriction* not dilation. This highlights not only a potential for separate pupil mechanisms during encoding versus retrieval but also the importance of the particular experimental context for determining whether pupil constriction or dilation occurs.

Although both the Kafkas and Montaldi (2015) and Naber et al. (2013) studies mentioned earlier are consistent in showing greater pupil dilation to familiarity during an encoding task, there are exceptions to this finding. In Laeng et al. (2007), three patients were tested using a similar memory paradigm as the aforementioned studies—with encoding and retrieval phases. Interestingly, even though the patients lacked explicit awareness of having seen the images before (failure of encoding due to hippocampal lesions), they still produced a greater pupil dilation to the novel *foil* stimuli during retrieval compared to previously seen stimuli, contrary to the pupillary response observed in the studies examining healthy participants. This further supports the notion that separate neural processes underpin encoding versus retrieval, and if damaged, the pupil will respond accordingly based only on the information sustained by the remaining pathways.

Many other studies have shown that stimulus novelty is capable of inducing a pupil dilation response (Ferrari et al., 2016; Kamp & Donchin, 2015; Kloosterman et al., 2015; Liao, Yoneya, Kidani, Kashino, & Furukawa, 2016). For example, Ferrari et al. (2016) found a greater dilation response for emotional compared to neutral natural scenes but only when they were novel; emotional content did not induce a greater pupil dilation when the scenes were repeatedly presented during the experiment. This highlights how a physiological response to novelty versus familiarity can be affected by other factors, such as arousal. In two studies concerning illusory phenomena, Kloosterman et al. (2015) and Beukema, Olson, Jennings, and Kingdom (2017) show that physically present and static stimuli can cause a pupil dilation when the images are perceived to vanish or move respectively, contrary to the physical reality.

The perception of illusory phenomena is a novel perception that has close ties with the concept of surprise—the betrayal of an individual’s level of certainty. Two studies that explore this concept more closely employ a gambling task while measuring participants’ pupil responses. In an auditory gambling task, participants placed bets on which of two cards (ranging in value from 1 to 10) would be of a higher numerical value. After the *first card* was announced, a level of *certainty* was established, and a greater pupil dilation response is observed for more certain outcomes (i.e., if the first card is 2, certainty is high that the second card will be of higher value). After the *second card* was announced, a level of *surprise* was established, and greater pupil dilation is observed for more surprising outcomes (i.e., if the first card was 2, but the second card was 1; Preuschoff, Marius, Einhäuser, & Nieuwenhuis, 2011). In another study, participants took part in a visual Iowa Gambling Task during pupil measurements. Similar to Preuschoff et al. (2011), Lavín, Martín, and Jubal (2014) showed that pupil dilation changes were modulated by learned uncertainty and surprise. These findings are relevant in the context of object recognition and familiarity because some level of uncertainty is inherently present when viewing unfamiliar forms (particularly nonobjects) when intermixed with other identifiable objects—even in a passive task-independent paradigm such as ours.

Pupillometry studies using sounds rather than visual patterns have also revealed a role for novelty in producing pupil dilation. When listening to a series of tones (high vs. low pitch) presented repetitively, conscious reports of deviations to these sound patterns are accompanied by a pupil dilation, presumably brought on by the novelty of the mismatched tone. On the other hand, if a deviation to the pattern occurred but was *not* consciously reported by the participant, pupil dilation was *not* observed (Quirins, Marois, Valente, & Seassau, 2018). This is arguably in contradiction to the aforementioned Naber et al.’s (2013) study, in which forgotten scenes that were previously encoded produced a *constriction* not dilation response. That is to say, one study shows that consciousness is important in order for novelty to produce a pupil dilation (Quirins et al., 2018), while another posits that when novelty is simply a result of forgetting it produces a pupil constriction (Naber et al., 2013).

The aim of this study is to test whether familiarity or novelty per se elicits a change in the pupil response. Given that unfamiliar 3D structures have been shown using functional magnetic resonance imaging to activate the same lateral-occipital networks that common objects do (Malach et al., 1995), one strategy for determining how familiar and unfamiliar objects are processed is to compare them in the absence of any task-specific encoding requirements or restrictions. The pupillometry studies discussed earlier examined the memory of encoded objects and scenes, but the stimuli in their experiments had a baseline level of familiarity prior to their encoding. That is, even unknown objects and scenes are familiar enough simply due to their relation to a wider semantic network of identifiable items and places. Our goal is to test the perception of identifiable objects as a marker of familiarity and unidentifiable nonobjects (and noise) as a marker of unfamiliarity.

An important methodological issue arises when one wishes to compare familiar with nonfamiliar objects: Which stimulus features should be common to both types of objects in order to make the comparison valid? When we encounter new objects in daily life, they usually possess certain common features with objects that we are already familiar with, such as straight edges, curved contours, textured surfaces, or familiar colors. If one thinks of object recognition as the end point of a hierarchy of visual processing from low-level to high-level features (Riesenhuber & Poggio, 1999), one ideally wants to match the two types of object as closely as possible to equate their responses throughout the hierarchy until one reaches the stage at which the critical differences that distinguish the familiar from the nonfamiliar are processed. Unfortunately, the literature on object recognition is insufficient to determine exactly where this point lies, so we made an educated guess. While many studies have attempted to control stimuli as best as possible to achieve this end, conventional methods have typically destroyed the most basic of visual properties, for example, when *phase-scrambling* the image, which involves randomizing the phases of the image’s Fourier components (Malach et al., 1995). This makes it difficult to determine whether the observed differences between objects and nonobjects is relevant to object recognition per se, as opposed to relevant to only the initial stages of visual processing (Stojanoski & Cusack, 2014).

**Figure 1.**
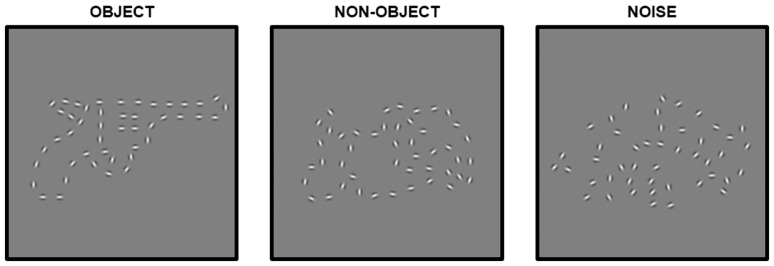
Example stimuli used in the experiments.

The stimuli used here, examples of which are shown in Figure 1, are designed to make as valid-as-possible a comparison between the pupil responses to familiar and unfamiliar objects. For example, an obvious prerequisite of any study using pupillometry to assess cognitive differences between conditions is that the stimuli across all conditions are equal in mean luminance; otherwise, one might simply be measuring the pupillary reflex to differing light levels. Our stimuli fulfil this requirement through the use of direct current-balanced Gabor micropatterns. The use of a fixed number of micropatterns also equates the stimuli for another low-level feature: average contrast content. The other manipulations aimed at matching the familiar with unfamiliar objects are described in the next section.

Based on findings that the pupil response is affected by perceptual content, arousal, and cognitive load (Bradley, Miccoli, Escrig, & Lang, 2008; Kloosterman et al., 2015; Peysakhovich, Dehais, & Causse, 2015), we expect to find pupil size differences between our familiar objects, the unfamiliar nonobjects, and the noise conditions. On the one hand, familiar objects evoke a semantic and possibly affective response, which previous studies suggest should cause an increase in pupil size (Partala & Surakka, 2003). On the other hand, more cognitive effort might be expended in processing nonfamiliar objects precisely because they are not familiar, or because they evoke more curiosity and arousal, and this might predict increased pupil size for nonfamiliar objects (Klingner, Tversky, & Hanrahan, 2011; Naber et al., 2013). The aim of this study is to test between these possible outcomes.

## Methods

The methods reported here are similar to those reported by Beukema et al. (2017). The procedure and analyses were preregistered online (see: https://osf.io/2wf3d/).

### Participants

Thirty healthy volunteers at McGill University were recruited as observers. No participants were excluded. The participants ranged in age from 18 to 40 years (median = 21.5; 19 females and 11 males). All participants had normal or corrected-to-normal visual acuity and did not possess any abnormalities of the eye. Demographic information about participants’ sex, age, ethnicity, handedness, eye color, and vision correction were collected. Participants gave informed consent prior to the study, and the research protocol was approved by the Research Ethics Board of the Research Institute of the McGill University Health Centre. Furthermore, this research adhered to the relevant tenets of the Declaration of Helsinki.

### Equipment

Stimuli were generated within the MATLAB Psychophysics Toolbox 3.0 and were displayed on a 1920 by 1080 pixel ASUS high-definition monitor. An EyeTribe pupilometer was used to collect eye gaze and pupil size data via infrared reflections using the EyeTribe toolbox for MATLAB (version 0.0.3; Dalmaijer, 2014). The EyeTribe sampled pupil size data at 60 Hz, collecting 540 data points per trial. Viewing distance to the monitor was 60 cm. Participants were placed in a chin and head rest to remove motion artifacts. Eye gaze and fixation were calibrated prior to each experimental block.

### Stimuli

The stimulus set was taken from a previous experiment investigating luminance inputs to mid- and high-level vision (Jennings & Martinovic, 2014). Jennings and Martinovic (2014) created their object stimulus set by choosing nameable line drawings of familiar objects from various stimulus sets (Alario & Ferrand, 1999; Bates et al., 2003; Hamm & McMullen, 1998) and digitally replacing the lines with Gabor patches that defined the outline shapes (Figure 1, first panel). The nonobjects were created by manipulating the distribution of the object-line drawings using image processing software and then applying the same shape-defining procedure as mentioned earlier to the subsequent Gabor placements (Martinovic, Mordal, & Wuerger, 2011). Importantly, the manipulation of objects into nonobjects attempted to preserve image complexity (same number of elements), aspect ratio (field of view), and closed outer shape contour (global structure), so that the resulting unfamiliar shapes retained comparability to their former objecthood (Figure 1, middle panel). All of the objects used in the current study were accurately identified with an accuracy rating of over 75% from all participants in Jennings and Martinovic (2014). A third stimulus group composed of random clusters of Gabor patches acted as a control (Figure 1, third panel). All conditions contained the same number of local Gabor patches, and all images in one condition were paired to an image of equal number of Gabors in the other conditions.

### Procedure

**Figure 2.**
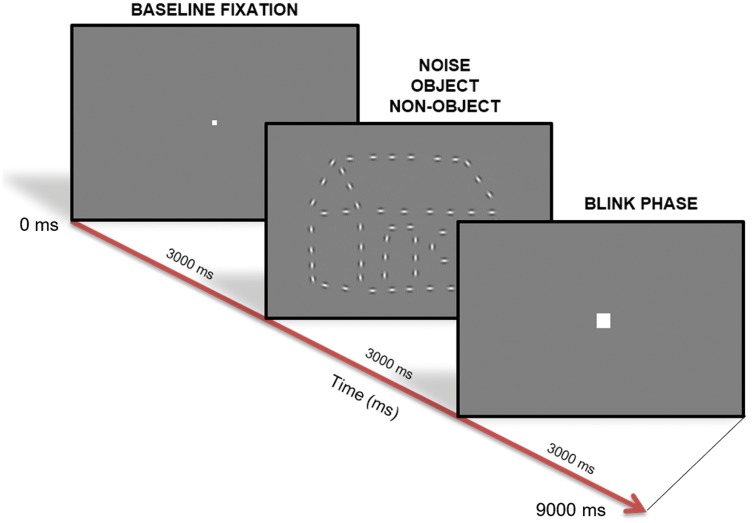
Experimental design. The three phases of a trial were the baseline fixation, the stimulus phase (noise, object, or nonobject conditions), and the blink fixation (3,000 milliseconds between each phase). Participants were instructed to fixate throughout the experiment and to attempt to isolate any blinks to the blink fixation phase. Example stimulus enlarged for clarity. All phases were 3 seconds in duration, for a trial length of 9 seconds, and a block length of 4.5 minutes.

Participants were shown sample trials from all three conditions prior to the experiment to ensure they could quickly and accurately identify whether the stimulus resembled an object, a nonobject, or a random array of Gabor elements. The actual experiment consisted of 50 images from each condition (150 total), broken up and randomly distributed into five 30-trial blocks to reduce dry eyes from visual fatigue. Each trial consisted of three phases: baseline, stimulus, and blink phases, as shown in Figure 2. The baseline phase served as a break from the stimulus to collect data for baseline correction. The phase of interest to the investigation is the stimulus phase during which pupillary differences were expected. Following this, participants were encouraged to blink a few times to reduce dry eyes, as blinks during the stimulus phase were discouraged in order to prevent data loss during the period of interest. Relatively long presentation times were used to ensure the perception of the stimulus was properly registered and ruminated, the after-image from the stimulus vanished, and that participants had ample time to blink. To avoid any effect of eye-movements on pupil size, participants were given a head rest for their chin and forehead and asked to remain fixated on the center of the screen for the entire length of the experiment.

### Data Processing and Analysis

Our data transformations mirror those of our previous study (Beukema et al., 2017, p. 6). The participants’ data were grouped and all trials were subject to postexperimental processing. Due to the high amount of noise inherent in pupillometry data, and the large amount of data collected at the 60 Hz sampling rate (540 samples per trial), samples within trials were excluded from the analysis according to two criteria: overly large fixational eye movements and blinks. To remove samples involving overly large fixational eye movements, coordinates outside of a 100-pixel radius from the fixation point for at least 15 samples (0.25 seconds) were rejected. To remove blink trials, if more than 10 contiguous samples (0.17 seconds) contained no data, a blink was assumed and the trial was excluded. Finally, if the stimulus phase contained fewer than 100 samples (1.7 seconds), the whole trial was excluded due to insufficient data. After all exclusions, 70.17% of the samples remained; all data are available online (https://osf.io/ve8kr/).

Data were baseline-corrected to the median pupil size within the last 200 milliseconds of the baseline phase to normalize the data. Statistical analyses focused on the segment of the stimulus phase occurring after the re-orienting pupil constriction caused by the onset response due to the sudden change in spatial frequency from blank fixation (Ebitz & Moore, 2019); analysis of the pupil time-course occurred from minimum pupil size in the stimulus phase (4,000 milliseconds) to the start of the blink phase (6,000 milliseconds).

We predicted that pupil size would vary by condition. To test this, we used linear regression to compare the average baseline-corrected pupil sizes for each condition, block, and participant. Using *t* tests with contrasts, we compared all three possible pairs of conditions. Tests were nondirectional with a Type I error rate of 0.05 and no family-wise error control. Assumptions were reasonable for all tests. For effect sizes, we use *d_R_*, a robust version of Cohen’s *d*, which shows standardized mean differences (Algina, Keselman, & Penfield, 2005). Square brackets throughout denote 95% bootstrapped confidence intervals. Data processing and analysis were done using R version 3.3.2 with packages multcomp, bootES, and ggplot2. All data are available online.

## Results

**Figure 3.**
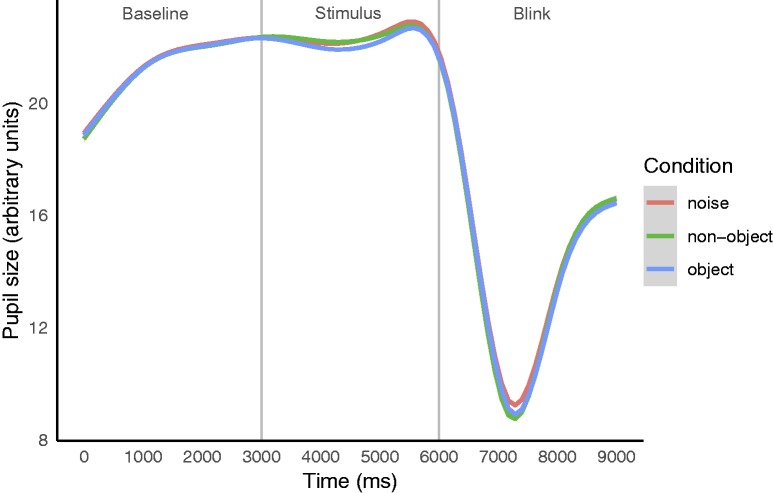
Averaged raw data showing pupil size by condition across the full trial. Pupil size is plotted as a function of time in milliseconds. Vertical lines represent onset and offset of phases outlined in Figure 2: baseline fixation from 0 to 3,000 milliseconds, stimulus (object, nonobject, noise) fixation phase from 3,000 to 6,000 milliseconds, and then blink fixation from 6,000 to 9,000 milliseconds. The small gray surrounds represent 95% confidence intervals. Lines show GAM-smoothed curves, as in subsequent plots.

**Figure 4.**
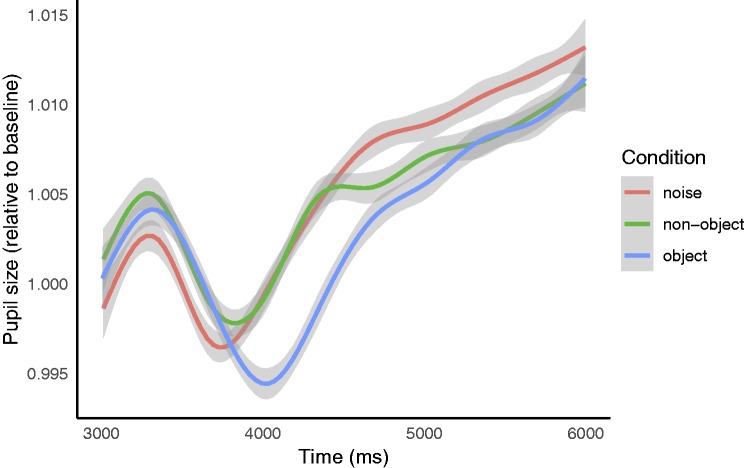
Baseline-corrected pupil size by condition. Expanded and processed data from the middle portion of Figure 3 (after baseline correction and artifact rejection), revealing the differences between conditions. The object stimulus (blue line) constricts pupils more than the noise (red line) and nonobject (green line) following the pupillary light reflex after stimulus onset. The gray surrounds represent 95% confidence intervals.

Figures 3 and 4 show, respectively, the pupil responses across the full trial duration (0–9,000 milliseconds) and across a portion of the trial duration (3,000–6,000 milliseconds). When averaged across the full stimulus phase (4,000–6,000 milliseconds), there was no difference in pupil size when viewing objects (*M* = 22.852 arbitrary units), nonobjects (*M* = 22.92), and random noise (*M* = 22.948). In line with previous studies measuring the pupil responses, we conducted a post hoc analysis over a narrower time window (4,000–4,500 milliseconds) corresponding to the fast reaction times found for discriminating static visual target stimuli (Barbur, Wolf, & Lennie, 1998; Kafkas & Montaldi, 2015; Naber et al., 2013; Schröger & Widmann, 1998). Within this stimulus range following the pupillary light reflex, pupil size was smallest when viewing objects (*M* = 22.689 arbitrary units) and of equal size when viewing nonobjects (*M* = 22.849) and random noise (*M* = 22.847; Figures 3 and 4).

## Discussion

The majority of studies investigating cognitively mediated pupil responses show a dilation response. Factors that have been shown to influence pupil dilation include anxiety, stress, recollection, cognitive effort, perceptual load, physical attraction, general emotional arousal (positive or negative), and novelty (Sirois & Brisson, 2014). As a result, the field of pupillometry is currently evolving to determine how the magnitude of pupil dilation is affected by these various factors.

In the present study, we investigated the effect of object form on pupil response, of which two competing hypotheses were proposed: First, that a greater dilation to familiar objects would be observed (given previous results showing pupil dilation to affective factors such as those elicited by familiar objects), and second, that a greater dilation to unfamiliar stimuli would be observed (given previous results showing pupil dilation to novelty). Our results showed that the unfamiliar stimuli (nonobject and noise conditions) provoked the greatest dilation when compared to the familiar object stimuli.

The effect of interest was significant only during a shorter time-window in a post hoc analysis (Table 1). This is not terribly surprising given that the methods were borrowed from our previous paper where the effect of interest was an illusion that physically persisted for the entire 3-second stimulus phase (Beukema et al., 2017). Our current stimuli, however, are more in line with other pupillometry studies employing a static visual discrimination task (Kafkas & Montaldi, 2015; Naber et al., 2013); therefore, there is nothing of interest (like motion) to perceive beyond the first half of a second, and this is in keeping with the rapid pupillary response to stimulus onset (Barbur et al., 1998; Schröger & Widmann, 1998). The paradigm was kept the same both for methodological consistency as well as to provide a precautionary window in case the pupillary light reflex needed more time to resolve.

A generic problem with pupillometry is that one can never be sure that the cause of pupil dilation is due to the stimulus manipulation itself rather than simply a low-level arrangement of the stimulus parts. Our combined use of stimuli made from Gabor micropatterns, as well as the inclusion of the random noise condition, enabled us to make a comparison between familiar and unfamiliar objects that minimized the effects of low-level stimulus factors. For example, there were inevitably differences in the number of contours between the nonobject and object conditions that could potentially cause the difference in pupil dilation. However, if the number of contours was critical, we would expect a difference between the nonobject and random-noise conditions, as the latter contain no contours; we found no difference between these two conditions.

The current results are intriguing due to the type of comparison being made. In Kafkas and Montaldi’s (2015) study, which showed greater pupil dilation in participants observing *old* objects previously seen compared to *novel* objects, all of the stimuli were selected from a familiar *class* of objects even though the specific instance of the object was novel. In our study, on the other hand, a *true* familiarity response was assessed as we compared familiar objects to never-before-seen nonobjects, even though the nonobjects comprised basic object properties.

There are at least two possibilities for why our unfamiliar conditions produced more dilation than the familiar objects, and they are not mutually exclusive: novelty and cognitive effort. Regarding novelty, the less interesting explanation is that the unfamiliar nonobjects and the random noise were simply more interesting than the familiar objects. However, this explanation is unlikely because objects inevitably elicit physiological arousal. Indeed, it may well be the case that our object condition elicited an arousal response, but that this response was mitigated by the fact that cognitive demands take priority over arousal in affecting the pupillary response (Stanners, Coulter, Sweet, & Murphy, 1979); that is, a pupil dilation response reflecting arousal is more likely when the cognitive demands are minimal.

Regarding cognitive effort, familiar objects may have a rich semantic network relevant to cognitive processing; however, recognition of the object itself is effortless. That is, it is likely that under the passive viewing conditions, participants viewed the objects more reflexively due to their high familiarity, while the unfamiliar conditions required a heightened level of cognitive effort before dismissing the stimuli as nonobjects. This theory would fit under the tonic activation profile of the locus coeruleus associated with task disengagement and encouraging exploration (Gilzenrat, Nieuwenhuis, Jepma, & Cohen, 2010).

**Table 1.** Pairwise Contrasts of Baseline-Corrected Pupil Size Between Conditions.

Range	Comparison	Difference	*t*	*p*	*dR*
4,000–6,000 milliseconds	Nonobject vs. Object	0.00202	0.736	.461	–0.06 [–0.14, 0.03]
	Noise vs. Object	0.00273	0.999	.318	–0.05 [–0.13, 0.04]
	Noise vs. Nonobject	0.00071	0.259	.795	0.01 [–0.07, 0.10]
4,000–4,500 milliseconds	Nonobject vs. Object	0.00629	2.575	.010	–0.11 [–0.20, –0.02]
	Noise vs. Object	0.0058	2.384	.017	–0.09 [–0.17, –0.01]
	Noise vs. Nonobject	–0.00049	–0.2	.841	0.03 [–0.06, 0.12]

*Note.* There was no difference when averaging across the full-time range (4,000–6,000 milliseconds, confirmatory test), but pupil size was smallest when viewing objects in a particular time range (4,000–4,500 milliseconds, post hoc test). *d_R_* shows robust Cohen’s *d* with bootstrapped 95% confidence intervals.

It is also worth considering that the result we are observing is due to the specific context of our experimental protocol, in which we compare three types of stimuli: familiar-object, unfamiliar-nonobject and unfamiliar-noise. For example, if the experiment were to test between two unfamiliar conditions, we might see a dilation to one over the other based on the stimulus properties. However, despite not giving the participants a task, there is likely still an implicit task with identifying objects from the other conditions and cognitive effort might therefore be invoked during the unfamiliar conditions in this experiment.

The current investigation outlines the importance of context within an experimental paradigm. Alone, the words *familiarity* and *novelty* are not enough to assign to a pupillary mechanism. In previous studies, these words were used to describe the stimuli *within* the experiment, categorizing remembered objects as familiar, and new or forgotten objects as novel. In our study, we use these terms more generally, categorizing all objects as familiar on the basis that they are recognizable despite never being observed before, and instead only using the word *novel* for unrecognizable forms with no identity. The difference in observed results between these two classifications further demonstrates a need for careful clarification in the study of cognitively mediated pupil size responses.
